# Knowledge, Attitude, and Practice of Weaning among Mothers in Najran Region, Saudi Arabia, 2021

**DOI:** 10.1155/2022/6073878

**Published:** 2022-03-02

**Authors:** Musalli Ali Al-Gashanin, Eisa Yazeed Ghazwani

**Affiliations:** ^1^Hai Al Dhubbat PHCC, The General Directorate of Health Affairs, Najran, Saudi Arabia; ^2^Family and Community Medicine Department, Faculty of Medicine, Najran University, Najran, Saudi Arabia

## Abstract

**Background:**

Weaning is a complex process of a gradual introduction of complementary foods to the infant's diet. It is recommended that solid food is introduced between 6 and 12 months of age. Weaning is difficult and potentially dangerous time for infants' growth and mother's education is an established determinant of its success. Little is known about weaning attitudes or practice among mothers in Najran Region of Saudi Arabia.

**Method:**

The study is a cross-sectional questionnaire-based observational investigation using random sampling scheme. We utilized ordinal logistic regression modelling to evaluate the relationship between demographic variables and knowledge level among mothers.

**Results:**

The total number included was *n* = 385 mothers who agreed to take part in the study. Good satisfactory knowledge rate was *n* = 135, 35.1%. Knowledge was mostly sought from other mothers (*n* = 102, 26.5%), followed by reliance on their own experience (*n* = 82, 21.3%). Seeking doctors' advice was associated with better knowledge levels. Worse adjusted knowledge scores were associated with bottle feeding (OR = 0.5383, *p*=0.0137), using cerelac preparations (OR = 0.0316, *p*=0.0092), development of weaning symptoms (OR = 0.5869, *p*=0.0260), seeking other mothers' advice (OR = 0.4750, *p*=0.0226), and feeding babies under 4 times daily (OR = 0.2742, *p*=0.0008). Mother education did not have significant impact on knowledge scores. *Discussion*. We confirmed, in this work, that knowledge levels were alarmingly unsatisfactory about weaning among our participants. Women were likely following local customs in terms of their choice of weaning methods even among the well-educated. The association between seeking doctors' advice and better knowledge should be utilized in future educational interventions. Underfeeding babies of below 4 times daily was correlated substantially with poorer knowledge score. This could be viewed as reverse causality, as clearly more knowledgeable mothers are expected to stick to optimum feeding frequency. *Recommendations*. Communication channels between physicians and mothers need to be opened and widened through focused educational programmes. Poor knowledge is clearly associated with infant underfeeding and difficulties in recognizing weaning symptoms. Such points need to be emphasized in design of health education packages to nursing mothers. Research on knowledge about weaning should focus on its association with traditional weaning methods and bottle feeding.

## 1. Introduction

The origins of the word ‘weaning' are traceable to the Anglo-Saxon expression “wenian” meaning “to become accustomed to something different” [1]. Weaning from breastfeeding is considered a natural and inevitable stage in the development of human child. Weaning is a complex process involving adjustment to a range of nutritional, immunological, biochemical, and psychological changes. Weaning may mean the complete cessation of breastfeeding (‘abrupt' or final wean) or, for the purposes of this paper, the beginning of a gradual process of the introduction of complementary foods to the infant's diet [2]. The very first introduction of foods other than breast milk is, by definition, the true beginning of weaning [2]. Breastmilk is the ideal food for infants [3]. It is safe and clean and contains antibodies which help protect against many common childhood illnesses. Breastmilk provides all the energy and nutrients that the infant needs for the first months of life, and it continues to provide up to half or more of a child's nutritional needs during the second half of the first year and up to one-third during the second year of life [4]. Breastfeeding without any supplementation (infant formula, water, and solid foods) is recommended for the first six months after birth [5]. Partial breastfeeding is recommended until the infant is at least 12 months old, and thereafter for as long as a woman and her child choose to continue. Partial breastfeeding is defined as breastfeeding while also providing other sources of nutrition, usually beginning at approximately six months of age. At this time, soft puréed meats, infant cereal, and then puréed fruits and vegetables may be introduced slowly. Cow's milk and fruit juice are not recommended until a child is at least 12 months old [6]. From a strictly nutritional perspective, weaning is the gradual process of transitioning infants from mother's milk to complementary foods and, ultimately, to an older child's diet. In this sense, weaning begins with the introduction of solids around the middle of the first year. Complete weaning, or complete cessation of breastfeeding, ideally should be a gradual process accomplished over a long period preferably baby-led [7].

Around the age of 6 months, an infant's need for energy and nutrients starts to exceed what is provided by breast milk, and complementary foods are necessary to meet those needs. An infant of this age is also developmentally ready for other foods. If complementary foods are not introduced around the age of 6 months, or if they are given inappropriately, an infant's growth may falter [8, 9]. A child of weaning age has a small stomach but needs plenty of food for growth and activity. There are two main ways of making sure these children get enough which is a very frequent feeding and using foods with a high concentration of nutrients. A very good first food to give a baby, along with breast milk, is a soft, thick, creamy porridge, made from the staple food of the community. Every community has a main staple food. It is often the first food that people think of when asked about their diet. The staple food contains starch, and it is eaten by most of the people in the community at most meals. It is usually less expensive than other types of food. The staple varies from country to country. It may be rice (for over 3 billion people around the globe [10]), wheat maize [11], cassava yam [12], and potato [13].

For the average healthy infant, meals should be provided 4–5 times per day, with additional nutritious snacks offered 1–2 times per day, as desired. The appropriate number of feedings depends on the energy density of the local foods and the usual amounts consumed at each feeding. If energy density or amount of food per meal is low, more frequent meals may be required [14].

There is a study in Saudi Arabia revealing that the mean age at supplementation with solid foods was 5.3 months. Literate mothers started supplementation with solids earlier than illiterate mothers [15].

The American Academy of Pediatrics (AAP) recommends feeding infants only breast milk for the first 6 months after birth. After 6 months, the AAP recommends a combination of solid foods and breast milk until the infant is at least 1 year old [16]. Weaning can be a dangerous time for babies. In many places, babies of weaning age do not grow well. They often fall ill and get more infections, especially diarrhoea, than at any other time. Babies who are malnourished may get worse during the weaning period and may become malnourished for the first time during weaning. Poor feeding and illness stop many children of weaning age growing well. This shows up on the growth chart as poor weight gain or, in more serious cases, as weight loss [17]. Education status of the mother has been identified as an important social determinant of health for children [18].

### 1.1. Primary Objective

It is to assess knowledge and to determine attitude and practice of mothers in weaning their children whose age ranges from four months to two years at Najran region in the year of 2021.

### 1.2. Secondary Objectives

1. To identify the relation between the mother's age and the weaning knowledge.
2. To detect the mean age of children when the weaning started.
3. To define the correlation between educational level of mothers and the weaning knowledge.
4. To identify the average times of children feeding per day.
5. To explore the relation between type of infants' family and weaning knowledge and practice of mothers.
6. To detect the type of food given to the child during weaning period.

## 2. Method

The current investigation was conducted utilizing a cross-sectional descriptive survey-based design and was run throughout the year 2021. We used a simple random sampling technique. There were 27 PHC centres all within Najran Health Directorate that were included in the full sampling frame. The sampling frame was a list of all infants attending the regular vaccination clinics, supplied with permission from the Information Technology Department in Najran Health Directorate. Pseudorandom numbers were created from random tables and used in all stages of sampling for the current study.

All mothers attended for vaccination clinics were eligible to be included in the current study. Exclusion criteria were being a mother of an infant who was either below four months or above two years. Mothers who were not fluent in Arabic language were also excluded from the survey. We excluded, in addition, cases where infants suffered from chronic conditions or serious congenital abnormalities.

Sample size calculations were performed to attain a power *β* = 0.8 and risk of type 1 error *α* = 0.05. Population in Najran region by gender and age group (female of age 15–49) in mid-2019 was 152614 according to the General Authority for statistics, Kingdom of Saudi Arabia. We included all the 27 PHCs in Najran. Utilizing the sample size calculator website (https://www.raosoft.com/samplesize.html), we needed to interview at least (*n* = 384) mothers, such that a power of 80% and significance level of 5% can be achieved.

The primary data collection tool was self-administered questionnaire given to mothers who attended the regular vaccination clinic with their infants. Mothers, who were sampled according to the pseudorandom number generator, were invited to participate by a trained research team member during their visit to the PHC and questionnaires were either handed over to them to fill using pen and paper or sent online to them to be filled electronically. All participants were given ample opportunity and encouraged to ask questions about the conduct of the study. Contact numbers for the principal investigator and the Research Ethics Committee were also given to mothers before a written consent was obtained. The questionnaire consisted of a section that included demographic data of the baby (such age, sex, and when weaning was commenced) and mother (age, nationality, residence, and count of children). It also included a section on weaning behaviour (such as what food was introduced and whether there were weaning-related symptoms). The questionnaire included a section on knowledge of mothers about weaning, such as what does weaning mean, duration of absolute breastfeeding, when to start weaning, and when it is allowed to give certain types of foods. Correct answers were added up and if more than half the score was attained (i.e., 3 out of five or more) that was taken as indication of ‘good knowledge'. We added a question on the source of weaning advice. The main items used in anaylsing the knowledge, attitude, and practice were as follows: “what does weaning mean exactly?” “What is the most suitable age for weaning your baby?” “What is the optimum duration for absolute breastfeeding?” “What do you consider the youngest suitable age for weaning?” “When do you think you can give honey to your baby?”

The face validity of the questionnaire was decided with a consensus of consultant family physicians practicing in child health in Najran area to whom we are very grateful. Internal consistency was measured using Cronbach's alpha estimate which was found to be 0.68 (within the acceptable range 60–70%).

Pilot study/pretesting: pilot study was carried on the 20 initial participants included. That was to evaluate appropriateness of data collection instrument, to identify the clarity and applicability of the tools and to provide feedback about the questionnaire and standardize the data collection approach. The principal investigator went through mothers' feedback and made alterations applicable to the questionnaire content accordingly.

### 2.1. Data Entry and Analysis

R-software was utilized in data analysis, table assembly, and graph design [19]. Categorical variables (such as educational level of mothers) were displayed as proportions, whereas continuous data (such as baby weight in kilograms) were presented in mean and standard deviation. Unadjusted analysis will be performed by comparing knowledge scores against each individual background factor using ordinal logistic regression modelling [20], which requires installation of the *MASS* package into the R-software canvas [21]. Knowledge score was collected from summing up the correct answers to five knowledge questions related to weaning. We also collected data on attitude and practice of weaning among mothers of children aging between 4 and 24 months in Najran city in the second half of 2021. Independent variables were age of mother, marital status of mother, number of children, education level of mother, and type of family. Adjusted analysis was carried out by constructing a full ordinal logistic regression model comprising of all the available factors and including, as predicted variable, the knowledge score of mothers [22]. Statistical significance cut-off was any probability value *p* < 0.05.

### 2.2. Ethical Considerations

Approval was sought and granted from Research Committee at the Ministry of Health Directorate in Najran. The research team started collecting the data using the questionnaire by direct interviewing the mother in private room. Written consent to participate in this study was ensured before commencing to fill the questionnaire. All data used during this research was ensured to be kept confidential and for the research purpose only. At the end of the study, a copy of the results and recommendations were submitted to the research committee institute.

## 3. Results

The total number of mothers included in the study was *n* = 385 mothers from Najran city.

For a detailed account of demographic results, see Table 1. The mean knowledge score was *μ* = 2.0 points (SD = 1.25 points), ranging between a minimum of 0 and a maximum score of 5 points. The median knowledge score was 2 points. Dividing the knowledge score to a good knowledge score if 3 points or over and poor knowledge if otherwise, we have the count of mothers with good knowledge to be *n* = 135, 35.1%. Mothers with poor knowledge about weaning were *n* = 250, 64.9%.

Among the mothers' babies who agreed to participate, there were *n* = 187, 48.6% baby boys and *n* = 198, 51.4% baby girls. There was no significant difference in terms of mothers' knowledge between male and female babies (*μ* = 2.0 and 2.1 respectively, *p*=0.8306).

**Figure 1:**
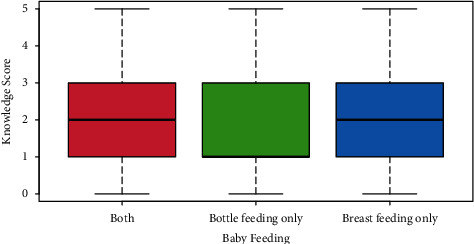
Mothers who bottle feed their babies (*n* = 103, 26.8%) scored far less (*μ* = 1.8 points, *p*=0.0013) in terms of knowledge score than breastfeeding women (*n* = 95, 24.7%, *μ* = 2.0 points, *p*=0.1505).

**Figure 2:**
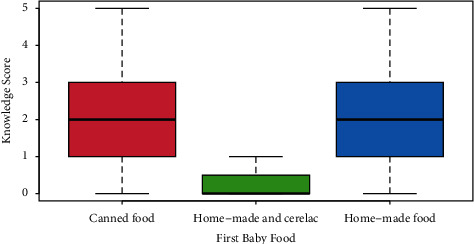
Compared to canned food (the red box-and-whiskers plot; *n* = 151, 39.2%, *μ* = 2.2 points), mothers who used home-made food and cerelac preparation scored the least knowledge score (the blue box-and-whiskers plot; *n* = 4, 1%, *μ* = 0.3 points, *p*=0.0013), whereas women who used home-made food only (the green box-and-whiskers plot) were majority (*n* = 230, 59.7%, *μ* = 1.9 points, *p*=0.0211).

In terms of baby age, there were *n* = 57, 14.8% who were between 4 and 6 months (scored mean knowledge *μ* = 1.9 points), with *n* = 89, 23.1% who were 19–24 months old and scored *μ* = 1.8 points. There were *n* = 67, 17.4% who were between 7 and 9 months old (*μ* = 2.2 points), in addition to *n* = 80, 20.8% who aged between 10 and 12 months and scored *μ* = 2.2 points. The 13–18 months old were *n* = 92, 23.9% and scored *μ* = 2.0 points. The difference was clearly nonsignificant in terms of average knowledge score (taking the 4–6 months old as reference group, mothers of 7–9-month-old babies had *p*=0.2434, whereas mothers of 10–12-month-old infants had *p*=0.2399, mothers of 13–18 months old had *p*=0.7654, and mothers whose babies were 19–24 months old had *p*=0.5111). Interestingly, as shown in Figure 1, mothers who bottle feed their babies (*n* = 103, 26.8%) scored far less (*μ* = 1.8 points, *p*=0.0013) in terms of knowledge score than breastfeeding women (*n* = 95, 24.7%, *μ* = 2.0 points, *p*=0.1505). Compared to canned food (*n* = 151, 39.2%, *μ* = 2.2 points), mothers who used cerelac preparation scored the least knowledge score (*n* = 4, 1%, *μ* = 0.3 points, *p*=0.0013), whereas women who used home-made food were majority (*n* = 230, 59.7%, *μ* = 1.9 points, *p*=0.0211). None of the demographic factors related to mothers was significantly associated with knowledge score, including mother education. See Figure 2.

**Figure 3:**
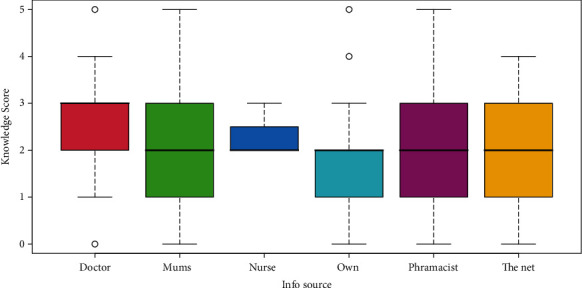
A visual display that consulting a doctor resulted in the highest knowledge score (red box) compared to other information source about weaning food choices.

In terms of seeking advice regarding weaning food, most participants would consult other mothers (*n* = 102, 26.5%), followed by reliance on their own experience (*n* = 82, 21.3%). However, as visualized in Figure 3, both categories were far less than those who consulted their doctors in terms of weaning knowledge (1.9 and 1,8 points respectively, compared to 2.5 points for those who sought advice from doctors; *n* = 57, 14.8%).

**Figure 4:**
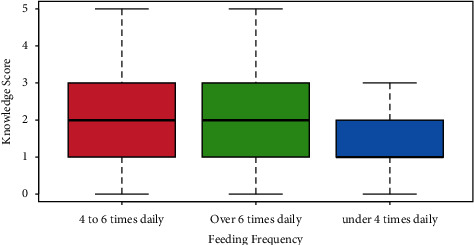
Most mothers reported feeding their babies between four and six times daily (scoring *μ* = 2.1 points in terms of average knowledge score). Those who fed their infants under four times daily score significantly lower knowledge score (*μ* = 1.2 points, *p* < 0.0001).

Regarding frequency of feeding, as on display in Figure 4, the majority of mothers (*n* = 329, 85.5%) reported feeding their babies between four and six times daily (scoring *μ* = 2.1 points in terms of average knowledge score). Those who fed their infants under four times daily scores significantly lower knowledge score (*n* = 31, 8.1%, *μ* = 1.2 points, *p* < 0.0001).

**Figure 5:**
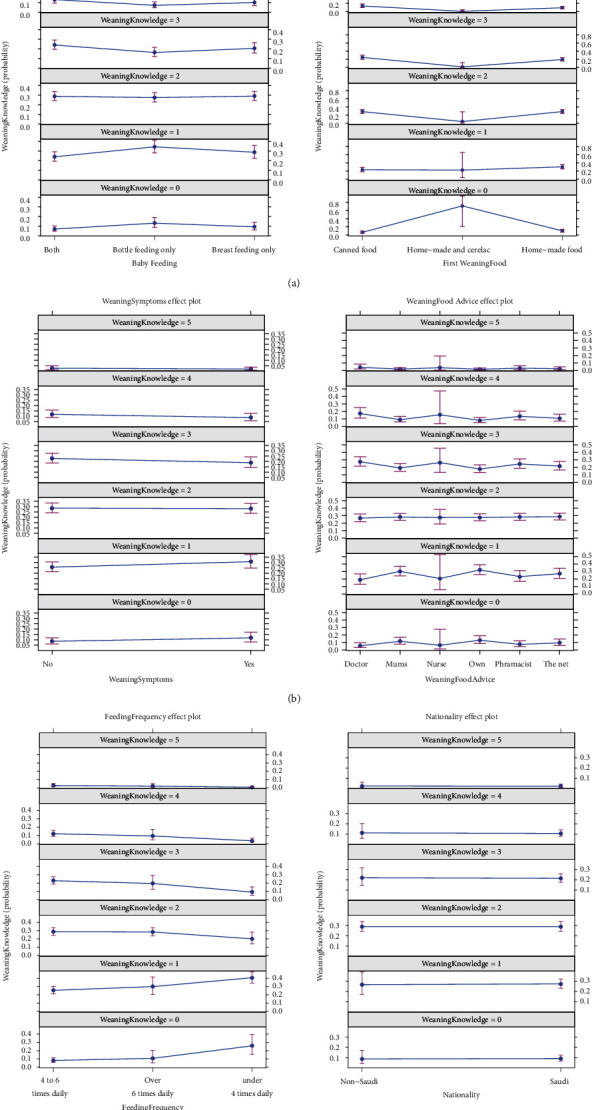
Estimates for the significant adjusted effects of clinical and demographic factors on knowledge score.

As detailed in Table 2 and Figure 5, the adjusted effects for demographic and feeding behaviour variables on the weaning knowledge score were assessed by fitting a multiple generalized linear model of the ordinal logistic type. A range of factors were found to impact the knowledge score significantly. Feeding a baby bottle only was associated with poorer knowledge score (OR = 0.5383, *p*=0.0137). Also using cerelac preparations were associated with substantially lower knowledge score (OR = 0.0316, *p*=0.0092). Having weaning symptoms was also associated with poorer weaning knowledge (OR = 0.5869, *p*=0.0260). Seeking weaning advice from other mothers led to far poorer knowledge score (OR = 0.4750, *p*=0.0226) as feeding babies under 4 times daily (OR = 0.2742, *p*=0.0008).

Figure 5 indicates that feeding a baby bottle only was associated with poorer knowledge score (OR = 0.5383, *p*=0.0137). Also using home-made food and cerelac preparations was associated with substantially lower knowledge score (OR = 0.0316, *p*=0.0092). Having weaning symptoms was also associated with poorer weaning knowledge (OR = 0.5869, *p*=0.0260). Seeking weaning advice from other mothers led to far poorer knowledge score (OR = 0.4750, *p*=0.0226) as feeding babies under 4 times daily (OR = 0.2742, *p*=0.0008).

## 4. Discussion of Key Findings

We investigated a sample of three hundred and eighty-five mothers in terms of knowledge, attitude, and practice of weaning their infants. Our survey is the largest of its kind in the south-west province in Saudi Arabia.

Satisfactory knowledge, as per our current findings, was poor among the surveyed mothers. Only over a third scored above 2 points (out of five points) in terms of knowledge of weaning. This is, of course, alarming. However, it is consistent with results of regional and global studies. A very recent Turkish investigation found that traditional methods of weaning are quite popular among mothers [23]. One earlier survey among Pakistani women indicated that self-reported knowledge among women regarding weaning was rated as 69% [24]. Poor knowledge clearly breeds unfavourable weaning practices. Women were impacted with local customs in terms of their choice of weaning methods for over three decades [25] and may be as long as humanity existed. In our current investigation, the majority declared reliance on their fellow mothers in choosing effective weaning food and method, with the poorest knowledge score though. Knowledge levels were suboptimum with regard to weaning even among working-class women [26]. One point of interest in our findings is the association between seeking doctors' advice and better knowledge score among our participants. Communication channels between physicians and mothers need to be opened and widened through focused educational programmes.

Regarding frequency of feeding, over 85% of mothers surveyed reported feeding their babies between four and six times daily. Furthermore, they were the most knowledgeable among our participants. We gather that they may have followed a baby-led feeding pattern. This was praised because of the perceived safety in terms of guarding against choking in young infants [27]. A notable minority among our participating women fed their infants under four times daily and, hence, scored a significantly lower average knowledge score. Underfeeding remains a substantial problem when weaning infants, particularly in rural communities [28]. On a positive note, recent estimates for frequency of complimentary feeding were regarded as satisfactory in rural India, exceeding 75% [29].

Many background factors played a significant role in terms of worsening knowledge levels among the women we included in the current investigation. Adopting bottle feeding was associated with lower knowledge scores. Unfortunately, bottle feeding is quite popular in developing nations; recent estimates amount to 77% engaging in such feeding practice [26]. That is clearly against the recommendations of healthcare professionals, of whom only over 3% advocated for baby-led weaning using bottle feeding [30]. The danger was the association between poor knowledge about weaning and bottle-feeding practice among our sample of mothers. Our results are clearly unique, as, to the best of our knowledge, no such association was reported before in the literature with this level of clarity.

Use of cerelac preparations did not improve overall knowledge as per our results. It was established over the last few decades that cerelac porridge was chosen by most women due to its attractive and tasteful flavours' range [31]. That was despite all the shortcomings of commercial food preparations [32]. Clearly higher knowledge about weaning food and weaning practice would deter women from using commercial preparations, such as cerelac. Such assertion was corroborated with the results of our study among Saudi mothers' weaning practices.

Among the mothers who participated in the current survey reports of weaning symptoms were much more among those with poorer weaning knowledge. This is clearly a unique finding of our research. None of the previous studies explored in sufficient detail the link between weaning symptoms and weaning knowledge. One would be prompted to hypothesize a potential association between over-recognition of weaning symptoms among mothers not knowledgeable enough to exclude such causation. However, such hypothesis requires reproduction by more focused research dedicated to exploring the ability of women to discriminate between weaning and nonweaning symptoms contrasted to their level of knowledge about the weaning process.

Additionally seeking conventional weaning knowledge of other peers did not improve knowledge outcomes among our sample of mothers. This provides further evidence to the noneffectiveness of traditional passing of knowledge about weaning among Saudi mothers. Doctors and health care educators will need to provide accurate information to mothers and encourage them to pass it to their peers in the community. A huge task indeed!

Underfeeding babies of below 4 times daily was correlated substantially with poorer knowledge score. This could be viewed as reverse causality, as clearly more knowledgeable mothers are expected to stick to optimum feeding frequency. However, such assertion may not hold true in Saudi Arabia as previous authors were concerned about the huge knowledge-practice gap [33].

There are several points of strength for the current study. We have a large sample size. However, the cross-sectional design and social desirability bias remain potential limitations to be taken on board before any generalization of the results to be made.

Future research should focus on the relationship between knowledge and bottle feeding and use of commercial food in more depth. Qualitative design for exploring mothers' values and thoughts about weaning has not been tried before and would offer real expansion of the current body of knowledge.

## 5. Conclusion

We found knowledge levels about weaning were poor among mothers in Najran. Women were likely following local customs in terms of their choice of weaning methods even among the well-educated. We found clear association between seeking doctors' advice and better knowledge, which needs to be capitalized on in design and delivery of future educational interventions. Underfeeding babies of below 4 times daily was correlated substantially with poorer knowledge score. This could be viewed as reverse causality, as clearly more knowledgeable mothers are expected to stick to optimum feeding frequency.

### 5.1. Recommendations

1. Communication channels between physicians and mothers need to be opened and widened through focused educational programmes.
2. Poor knowledge is clearly associated with infant underfeeding having difficulties in recognizing weaning symptoms. Such points need to be emphasized in design of health education packages to nursing mothers.
3. Research on knowledge about weaning should focus on its association with traditional weaning methods and bottle feeding.

## Figures and Tables

**Table 1:** Baseline demographics of mothers and unadjusted effect on knowledge score.

Factor	Count (n)/mean (*μ*)	Percentage/SD	Mean knowledge score	*P* value
Baby age				
4–6 months	57	14.8%	1.9 points	Reference
7–9 months	67	17.4%	2.2 points	0.2434
10–12 months	80	20.8%	2.2 points	0.2399
13–18 months	92	23.9%	2.0 points	0.7654
19–24 months	89	23.1%	1.8 points	0.5111
Baby gender				0.8306
Male	187	48.6%	2.0 points	
Female	198	51.4%	2.1 points	
Baby weight	*μ* = 9.8 kg	SD = 3.4 kg	*r* = 0.0230	0.422
Baby feeding				
Breastfeeding	95	24.7%	2.0 points	0.1505
Bottle feeding	103	26.8%	1.8 points	0.0013
Both	187	48.6%	2.2 points	Reference
Nonmilk food				
Yes	365	94.8%	2.05 points	0.6926
No	20	5.2%	1.95 points	Reference
Feeding start age	*μ* = 5.3 months	SD = 2.3 months	*r* = −0.0989	0.0761
Weaning food				
Home-made food	230	59.7%	1.9 points	0.0211
Cerelac and home	4	1.0%	0.3 points	0.0013
Canned food	151	39.2%	2.2 points	Reference
Mixed food				
Yes	275	71.4%	2.0 points	Reference
Single-type food	110	28.6%	2.2 points	0.2707
Weaning symptom				
Yes	114	29.6%	1.8 points	0.03524
No	271	70.4%	2.1 points	Reference
Food choice				
Doctor	57	14.8%	2.5 points	Reference
Nurse	3	0.8%	2.3 points	0.8413
Pharmacist	6	16.9%	2.3 points	0.3557
Other mothers	102	26.5%	1.9 points	0.0042
Own experience	82	21.3%	1.8 points	0.0023
Internet	7	19.7%	2.0 points	0.0519
Feeding frequency				
Under 4 daily	31	8.1%	1.2 points	<0.0001
4–6 daily	329	85.5%	2.1 points	Reference
Over 6 daily	25	6.5%	1.9 points	0.4068
Nationality				
Saudi	361	93.8%	2.04 points	0.9862
Non-Saudi	24	6.2%	2.00 points	Reference
Mother age				
Under 20	8	2.1%	1.3 points	0.0535
20–24	81	21.0%	2.1 points	Reference
25–29	121	31.4%	2.1 points	0.9056
30–34	95	24.7%	2.1 points	0.8817
35–40	75	19.5%	1.9 points	0.1356
Over 40	5	1.3%	1.2 points	0.0772
Mother education				
Illiterate	6	1.6%	1.3 points	0.2213
Intermediate	11	2.9%	2.1 points	Reference
Primary	4	1.0%	2.5 points	0.5462
Secondary	185	48.1%	2.2 points	0.7331
University	179	46.5%	1.9 points	0.5561
Mother marital				
Divorced	20	5.2%	2.4 points	Reference
Married	359	93.2%	2.0 points	0.3258
Widowed	6	1.6%	1.8 points	0.4091
Residence				
Children and relatives	2	0.5%	1.0 points	Reference
Children only	21	5.5%	2.3 points	0.1336
Children and husband	226	58.7%	2.0 points	0.2287
Children, H and relatives	136	35.3%	2.1 points	0.1997
Kids count	2.4 kids	SD = 1.4 kids	*r* = −0.0633	0.2107

**Table 2:** Description of the unadjusted effects for demographic factors on weaning knowledge.

Factor	OR	95% CI of OR	*P* value
Kids count	0.9462	0.7389 to 1.2117	0.6613998
Baby age: 19–24 months	0.5762	0.1705 to 1.9478	0.3756812
Baby age: 7–9 months	1.3450	0.6212 to 2.9123	0.4525357
Baby age: 10–12 months	0.9346	0.4029 to 2.1677	0.8748855
Baby age: 13–18 months	0.7877	0.2994 to 2.0725	0.6291193
Baby sex: male	1.1482	0.7744 to 1.7024	0.4922074
Baby weight	1.0776	0.9603 to 1.2094	0.2047260
Baby feeding: bottle only	0.5383	0.3299 to 0.8783	0.0136594^*∗*^
Baby feeding: breast only	0.6068	0.3684 to 0.9994	0.0505671
Food other than milk	2.6006	0.2923 to 23.1363	0.3920445
Age started feeding	0.9326	0.8498 to 1.0234	0.1420095
Weaning food: cerelac	0.0316	0.0024 to 0.4185	0.0091932^*∗∗*^
Weaning food: home-made food	0.5687	0.3705 to 0.8731	0.0103101^*∗*^
Simple Single-type food	1.1825	0.7570 to 1.8474	0.4618558
Weaning symptoms	0.5869	0.3679 to 0.9364	0.0260458^*∗*^
Weaning advice: nurse	0.3047	0.0219 to 4.2489	0.3773823
Weaning advice: pharmacist	0.5396	0.2618 to 1.1122	0.0955201
Weaning advice: mothers	0.4750	0.2512 to 0.8982	0.0226459^*∗*^
Weaning advice: own experience	0.6280	0.3185 to 1.2384	0.1802782
Weaning advice: Internet	0.5497	0.2841 to 1.0636	0.0765412
Feeding: > 6 daily	1.2169	0.5092 to 2.9080	0.6590821
Feeding: > 4 daily	0.2742	0.1301 to 0.5778	0.0007519^*∗∗∗*^
Mother age: 25 to 29	1.0551	0.5750 to 1.9360	0.8626867
Mother Age:30 to 34	1.3275	0.6165 to 2.8585	0.4696366
Mother Age:35 to 40	0.8386	0.3203 to 2.1956	0.7201903
Mother Age:> 40	1.1116	0.0791 to 15.6302	0.9375196
Mother Age:< 20	0.6501	0.0999 to 4.2309	0.6525491
Nationality: Saudi	0.8961	0.3531 to 2.2745	0.8176355
Education: none	0.1716	0.0196 to 1.5011	0.1121546
Education: primary	1.8978	0.1951 to 18.4581	0.5813017
Education: secondary	0.7064	0.1907 to 2.6159	0.6031459
Education: university	0.3833	0.1019 to 1.4415	0.1569112
Marital: married	0.6272	0.0413 to 9.5257	0.7370485
Marital: widowed	1.1792	0.1361 to 10.2164	0.8811800
Residence: children only	4.9000	0.0821 to 292.507	0.4467834
Residence: husband and children	3.8842	0.0274 to 549.897	0.5916521
Residence: husband kids and relatives	3.5073	0.0249 to 494.422	0.6195171

^*∗∗∗*^indicates statistical significance below the *p*=0.001 level. ^*∗∗*^indicates statistical significance below the *p*=0.01 level. ^*∗*^indicates statistical significance below the *p*=0.05 level.

## Data Availability

The datasets generated during and/or analyzed during the current study are available from the corresponding author on reasonable request.
